# Prediction of active drug-resistant pulmonary tuberculosis based on CT radiomics: construction and validation of independent models and combined models for residual pulmonary parenchyma

**DOI:** 10.3389/fmed.2025.1508736

**Published:** 2025-03-31

**Authors:** Mingke Liu, Yongxia Zhou, Jing Ding, Fuli Wei, Fang Wang, Siyao Nie, Xianv Chen, Yuting Jiang, Mingmeng Huang, Liangbo Hu

**Affiliations:** ^1^Department of Radiology, The Affiliated Yongchuan Hospital of Chongqing Medical University, Chongqing, China; ^2^Department of Infection, The Affiliated Yongchuan Hospital of Chongqing Medical University, Chongqing, China; ^3^Department of Research and Development, Shanghai United Imaging Intelligence Co., Ltd., Shanghai, China; ^4^Department of Preventive Medicine, The Affiliated Yongchuan Hospital of Chongqing Medical University, Chongqing, China; ^5^Department of Radiology, Beibei Hospital of Chongqing Medical University, Chongqing, China

**Keywords:** pulmonary tuberculosis, drug resistance, radiomics, machine learning, computed tomography (CT)

## Abstract

**Background:**

Drug-resistant tuberculosis (DR-TB) is a severe public health threat and burden worldwide. This study seeks to develop and validate both independent and combined radiomic models using pulmonary cavity (PC), tree-in-bud sign (TIB), total lung lesions (TLL), and residual pulmonary parenchyma (RPP) to evaluate their effectiveness in predicting DR-TB.

**Methods:**

We recruited 306 confirmed active pulmonary tuberculosis cases from two hospitals, comprising 142 drug-resistant and 164 drug-sensitive cases. Patients were assigned to five training and testing cohorts: PC (*n* = 109, 47), TIB (*n* = 214, 92), TLL (*n* = 214, 92), RPP (*n* = 214, 92), and their combination (*n* = 109, 47). Radiomic features were extracted using variance thresholding, K-best, and LASSO techniques. We developed four separate radiomic models with random forest (RF) for DR-TB prediction and created a combined model integrating all features from the four indicators. Model performance was validated using ROC curves.

**Results:**

We extracted 10, 2, 10, 3, and 9 radiomic features from PC, TIB, TLL, RPP, and the combined model, respectively. The combined model achieved AUC values of 0.886 (95% CI: 0.827–0.945) in the training set and 0.865 (95% CI: 0.764–0.966) in the testing set. It slightly surpassed the PC model in the training set (0.886 vs. 0.850, *p* < 0.05) and was comparable in the testing set (0.865 vs. 0.850, *p* > 0.05). The combined model showed similar performance to the TIB, TLL, and RPP models in both sets (*p* > 0.05).

**Conclusion:**

The newly defined and developed RPP model and the combined model demonstrated robust performance in identifying DR-TB, highlighting the potential of CT-based radiomic models as effective non-invasive tools for DR-TB prediction.

## 1 Introduction

Tuberculosis (TB), caused by *Mycobacterium tuberculosis* (MTB), is one of the infectious diseases that poses a significant threat to human health, contributing to a substantial global disease burden and mortality rate (1). The issue of drug-resistant tuberculosis (DR-TB) has become increasingly severe, presenting ongoing challenges to public health systems and necessitating more effective prevention and control strategies from global health organizations (1). DR-TB specifically refers to strains confirmed by laboratory drug susceptibility testing to be resistant to at least one first-line anti-tuberculosis drug, such as rifampicin or isoniazid (2). Its treatment is lengthy, complex, and costly, far exceeding that of drug-sensitive tuberculosis (DS-TB) (3). Timely diagnosis and early treatment of DR-TB are crucial for controlling its transmission and progression (4, 5), making it one of the core challenges in current prevention and control efforts (6).

Although traditional methods such as microbial culture and sputum smear microscopy are commonly used for TB diagnosis (2), these approaches are limited by the low sensitivity of sputum tests and lengthy culture periods—MTB results typically take 4–8 weeks to obtain (1, 7). Moreover, these methods cannot adequately cover cases with negative sputum cultures, leading to a risk of diagnostic omissions (3). Some tuberculosis patients without sputum or with insufficient sputum cannot be diagnosed through microbiological culture and sputum smear microscopy methods. In recent years, gene detection technologies like linear probe assays and whole-genome sequencing have emerged to shorten diagnosis time; however, they are constrained by the bacterial load in the sample and laboratory conditions (7). Therefore, there is an urgent need to explore a non-invasive and easily implementable method for accurately identifying and predicting the drug resistance status of pulmonary tuberculosis patients.

Currently, the application of artificial intelligence technology in the field of tuberculosis imaging is gradually emerging (8–10). Notably, radiomics—an innovative, non-invasive approach—can automatically and efficiently extract a substantial amount of quantitative information from specific regions of medical images (11). This provides robust data support for clinical decision-making, aiming to enhance diagnostic accuracy, prognostic assessment, and predictive capabilities. This technology has been widely utilized in differentiating between benign and malignant lesions, as well as in disease diagnosis and prognosis analysis (12–15). However, research on utilizing radiomics to predict drug resistance in active tuberculosis remains relatively limited (16).

In this study, we have innovatively introduced the concepts of “TLL” and “RPP” Given the diverse manifestations and widespread distribution of drug-resistant pulmonary tuberculosis in CT imaging, the development of the “TLL” model is particularly significant, offering a new perspective for deeper insights into the disease. Notably, the term “RPP” is proposed for the first time in this research, marking a substantial advancement in our exploration of radiomics in pulmonary tuberculosis. Historically, previous studies have concentrated on the typical CT presentations of DR-TB, with minimal focus on the imaging characteristics of normal or nearly normal lung tissue surrounding the tuberculosis lesions, referred to as “RPP.” This study addresses that gap.

Radiomics generally focuses on single lesions as the subject of study. However, tuberculosis lesions are diverse and widely distributed, with the main types including TIB, PC, consolidation, and fibrous strands, among which TIB and PC are the most common (17, 18). Studying these common different types of lesions can provide multiple perspectives for exploring the characteristics of DR-TB. Given this context, the current study seeks to develop a predictive model using CT imaging features such as pulmonary cavity (PC), tree-in-bud sign (TIB), total lung lesions (TLL), and residual pulmonary parenchyma (RPP). The goal is to validate the model’s ability to distinguish drug-sensitive tuberculosis (DS-TB) from DR-TB, thereby providing imaging evidence for the early identification and intervention of drug-resistant pulmonary tuberculosis in clinical practice.

## 2 Materials and methods

### 2.1 Patients

We collected clinical and imaging data from 306 confirmed cases of active pulmonary tuberculosis treated at two institutions between January 2016 and August 2023. The clinical data we collected for the patients included gender, age, and treatment history, and there was no statistically significant difference in clinical data between the two institutions. All data review and analysis phases of this study received formal approval from the Institutional Review Board, which waived the requirement for post-consent from participants in accordance with their guidelines.

**Inclusion criteria**: (a) Patients met the diagnostic criteria outlined in the World Health Organization’s Comprehensive Tuberculosis Guidelines (2); (b) Results of drug susceptibility testing (DST) for *Mycobacterium tuberculosis* were used to differentiate between DS-TB and DR-TB; and (c) Complete clinical and imaging examination records were available.

**Exclusion criteria**: (a) Poor image quality or incomplete clinical information; (b) History of other pulmonary diseases, such as lung cancer; and (c) Diabetes or HIV seropositivity.

**Figure 1:**
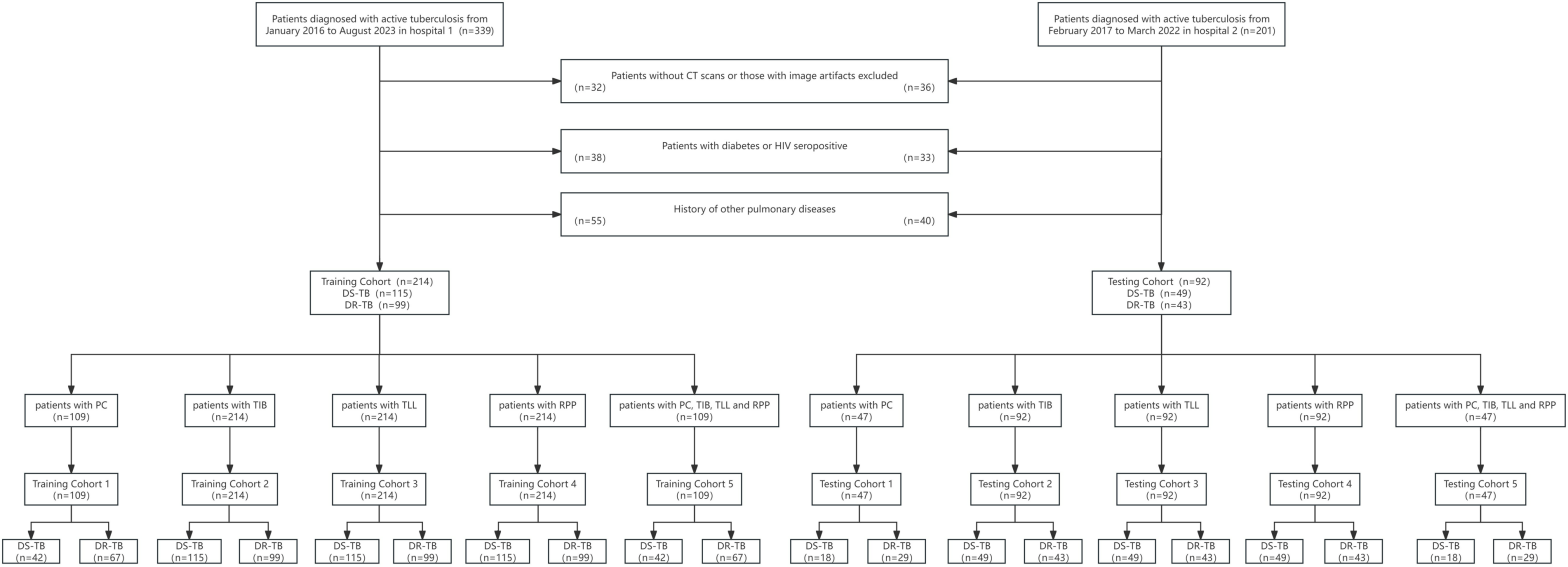
Flowchart of patient selection. PC, TIB, TLL, and RPP stand for pulmonary cavity, tree-in-bud sign, total lung lesions, and residual pulmonary parenchyma, respectively.

Ultimately, we enrolled 306 confirmed cases of active pulmonary tuberculosis from two hospitals between January 2016 and August 2023, encompassing 142 cases of DR-TB and 164 cases of drug-sensitive tuberculosis. Hospital 1 contributed 214 patients (115 DS-TB and 99 DR-TB), which comprised the training cohort, whereas Hospital 2 provided 92 patients (49 DS-TB and 43 DR-TB), forming the testing cohort, with a ratio of 7:3 between the training and testing cohorts. The patients were further categorized into five distinct training and testing cohorts based on their different imaging features: PC training cohort: 109 patients (42 DS-TB, 67 DR-TB); testing cohort: 47 patients (18 DS-TB, 29 DR-TB). TIB, TLL, and RPP training cohort: 214 patients each (115 DS-TB, 99 DR-TB); testing cohort: 92 patients each (49 DS-TB, 43 DR-TB). The combined model training cohort: 109 patients (42 DS-TB, 67 DR-TB); testing cohort: 47 patients (18 DS-TB, 29 DR-TB). Detailed information on the patient recruitment flowchart is presented in Figure 1.

### 2.2 Methods

All pulmonary CT scans were performed using the Philips Brilliance 256-slice iCT, SOMATOM go. Top CT systems, and SOMATOM Definition AS 128 CT. Patients were positioned supine and instructed to inhale and hold their breath, with the scanning range extending from the lung apices to the diaphragm. High-resolution computed tomography (HRCT) techniques were utilized, with the following parameters: tube voltage set to 120 kV, intelligent mAs, a rotation speed of 0.5 s per revolution, a pitch of 0.758, collimation of 128 × 0.625, slice thickness of 5 mm, and slice spacing of 5 mm. Thin-slice reconstructions were performed with a thickness of 1 mm and a spacing of 0.5 mm. Subsequently, the processed imaging data were imported into the “United Imaging uAI research portal 211230” platform for in-depth analysis of radiomic features.

### 2.3 Radiomics analysis

#### 2.3.1 Lesion segmentation

**Figure 2:**
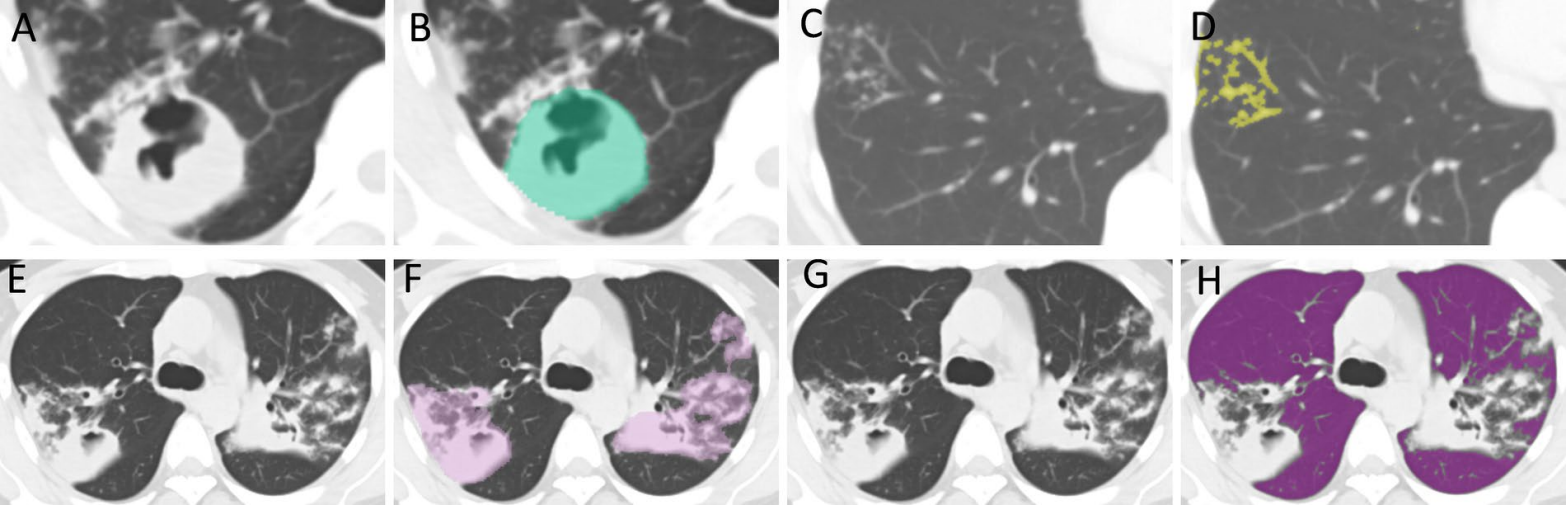
ROI Delineation. **(A,B)** The delineation of regions of interest (ROI) for PC. **(C,D)** The delineation for TIB. **(E,F)** The delineation for TLL. **(G,H)** The delineation for RPP. PC, TIB, TLL, and RPP stand for pulmonary cavity, tree-in-bud sign, total lung lesions, and residual pulmonary parenchyma, respectively.

CT images were analyzed blindly by an attending physician (Physician 1) and an associate chief physician (Physician 2), each with over 10 years of experience in radiology. The primary imaging features evaluated on the CT scans included: (A) TIB; (B) PC; (C) consolidation; (D) fibrous strands; (E) calcified nodules; (F) solitary large nodules with surrounding satellite lesions; and (H) caseous pneumonia. TLL related to tuberculosis were defined as all imaging manifestations confirmed to be associated with pulmonary tuberculosis, whereas RPP referred to the normal lung tissue in both lungs, excluding the TLL. Physician 1 obtained regions of interest (ROI) layer by layer along the edge of the largest lesion to segment PC, TIB, TLL, RPP (as shown in Figure 2). A combined approach of manual delineation and automatic segmentation was employed to delineate contours. After a 2-week interval, 50 cases were randomly selected for re-delineation of ROIs by both Physician 1 and Physician 2 using the same method. Both physicians remained blinded to the drug sensitivity test (DST) results to ensure objective evaluation.

#### 2.3.2 Extraction and selection of radiomic features

During the image preprocessing stage, the bin width was set to 25, and the image was resampled using BSpline interpolation to a voxel size of 1 × 1 × 1 mm. Subsequently, the original image underwent transformations through various filters, including Laplacian of Gaussian, Recursive Gaussian, Discrete Gaussian, and wavelet transform filtering. Radiomic features were extracted from PC, TIB signs, TLL, RPP, and combined lesion groups. A total of 2,264 radiomic features were derived from the lesion ROIs, which included 104 original image features and 2,160 filtered features, encompassing first-order features, shape features, and texture features. The combined lesion group includes features extracted from individual lesion models (PC, TIB, TLL, and RPP), rather than being based on a single combined ROI. Patients were allocated into training and Testing sets in a 7:3 ratio, with the PC model and combined model comprising training set (*n* = 109) and Testing set (*n* = 47). The TIB model, TLL model, and RPP model had patient groups of training set (*n* = 214) and Testing set (*n* = 92), using a random seed of 80. Z-score normalization was applied to mitigate dimensional discrepancies among the various radiomic features. The intra-class correlation coefficient (ICC) was utilized to select radiomic features demonstrating high consistency (ICC > 0.75) among observers. Optimal radiomic features were identified using the variance threshold method, Select K Best (with K set to 20), and the Least Absolute Shrinkage and Selection Operator (LASSO) algorithm.

#### 2.3.3 Model construction

The Random Forest Classifier (RFC) is a method known for its high variance-bias trade-off, making it an effective choice for constructing predictive models (19). To assess the performance of various models in predicting drug resistance in tuberculosis patients, the RFC method was utilized to train the selected radiomic features from PC, TIB, TLL, RPP, thus establishing individual models for each feature set. Ultimately, a combined model was developed, incorporating all radiomic feature combinations derived from the four previous models.

### 2.4 Statistical analysis

Statistical analyses were conducted using SPSS version 26.0 and R software version 3.5.0. Categorical data were expressed as “counts” and “proportions (%),” with the *χ^2^* test for *R × C* contingency tables employed to evaluate differences between groups. For normally distributed continuous variables, results were presented as “*x*±*s*,” and independent samples *t*-tests were conducted to assess group differences. In contrast, skewed continuous data were reported as median (IQR) [*M*(Q1-Q3)] and compared using the Mann–Whitney U test. Furthermore, the receiver operating characteristic (ROC) curve was utilized to assess the performance of the five models across the two independent cohorts. The DeLong test was employed to analyze the area under the curve (AUC) for the PC model, TIB model, TLL model, RPP model, and combined model. For each model, sensitivity, specificity, accuracy, and balanced F-score were calculated, with statistical significance set at *p* < 0.05.

## 3 Results

### 3.1 Clinical characteristics of patients

The clinical characteristics of the 306 patients are summarized in Table 1. Statistically significant differences were identified between DR-TB and DS-TB patients concerning gender (*p* < 0.001), age (*p* < 0.001), and treatment history (*p* < 0.001).

**Table 1:** Comparison of general data between two groups of patients with drug-resistant tuberculosis (DR-TB) and drug-sensitive tuberculosis (DS-TB).

	DR-TB (*n* = 142)	DS-TB (*n* = 164)	Statistical test value Z/χ^2^	*p*-value
Gender [cases, composition ratio (%)]			19.535	<0.001
Male	123 (86.6)	106 (64.6)		
Female	19 (13.4)	58 (35.4)		
Age [years, M (Q1, Q3)]	52.0 (43.8, 64.0)	36.5 (23.0, 57.0)	−5.347	<0.001
Treatment History [cases, composition ratio (%)]			51.576	<0.001
New treatment	85 (59.9)	154 (93.9)		
Re-treatment	57 (40.1)	10 (6.1)		

Gender: Composition ratios represent the percentages of male and female patients within each group.

Age: Data presented as median (interquartile range, IQR). Mann–Whitney U test was used for comparison.

Treatment History: Composition ratios represent the percentages of new treatment and re-treatment patients within each group. Chi-square test was used for comparison.

Statistical Test Value (Z/χ^2^): For gender and treatment history, χ^2^ (chi-square) statistic is reported. For age, Z-statistic from Mann–Whitney U test is reported.

*p-value: Indicates the statistical significance of the comparison between the two groups. p-values less than 0.05 indicate statistically significant differences.*

### 3.2 Radiomic feature selection

**Figure 3:**
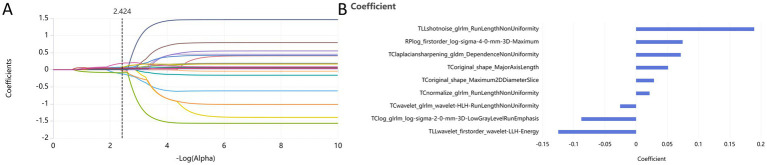
Dimensionality reduction of radiomics features for the combined sign group using the LASSO algorithm **(A)** and retained features after screening **(B)**. PC, TIB, TLL, and RPP stand for pulmonary cavity, tree-in-bud sign, total lung lesions, and residual pulmonary parenchyma, respectively.

Following the screening of the extracted 2,264 radiomic features, the optimal features were selected for the following groups: PC group, TIB group, TLL group, RPP group, and combined sign group. The retained features were as follows: the PC group retained 10 features (2 shape features and 8 texture features); the TIB group retained 2 features (both texture features); the TLL group retained 10 features (4 first-order features and 6 texture features); the RPP group retained 3 features (2 first-order features and 1 texture feature); and the combined sign group retained 9 features (2 shape features, 2 first-order features, and 5 texture features). The radiomics features of the combined sign group selected by LASSO are illustrated in Figure 3.

### 3.3 Model performance and validation

**Figure 4:**
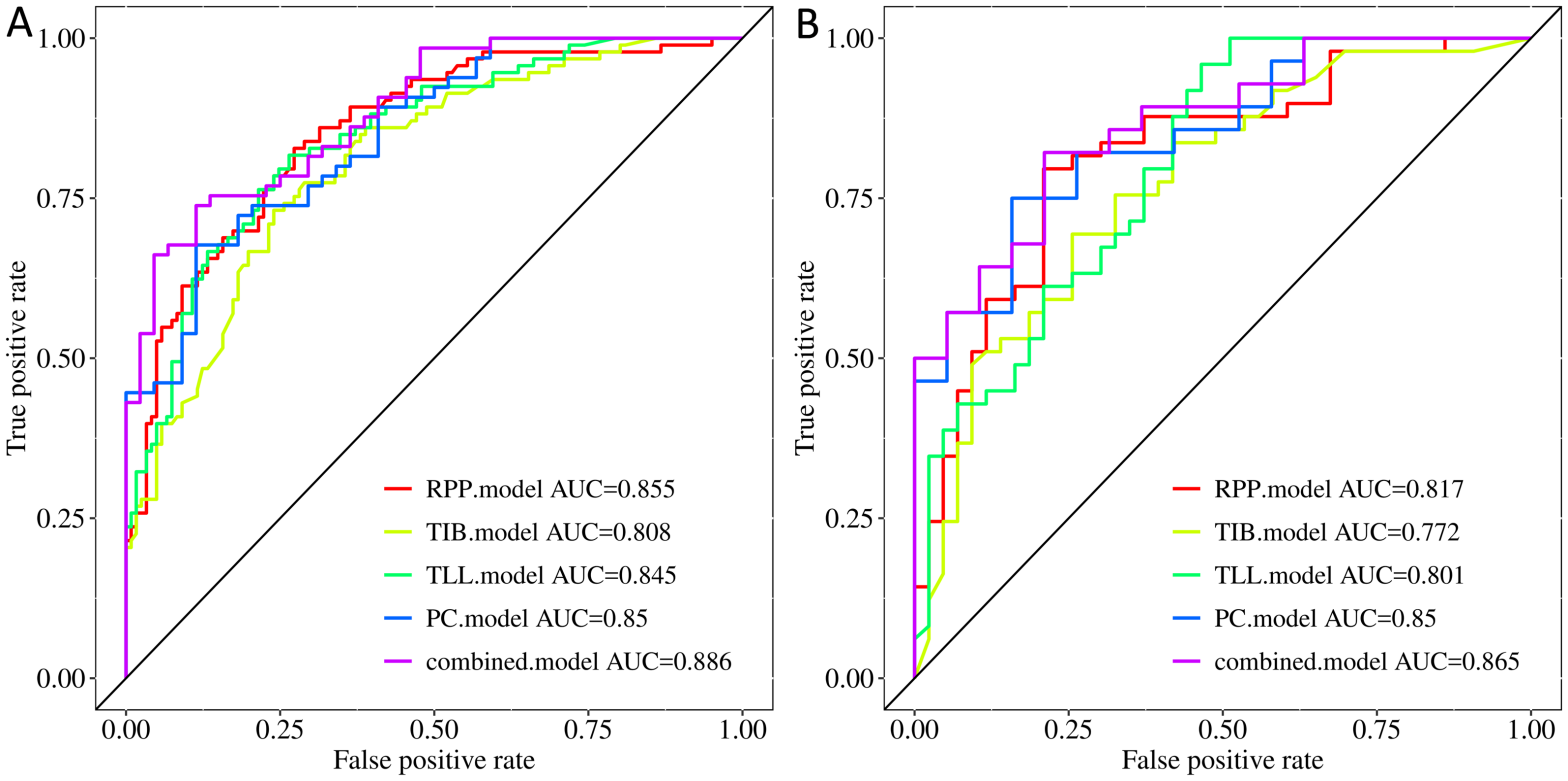
ROC curves of the PC, TIB, TLL, RPP, and combined model. **(A)** Training Set. **(B)** Testing Set. PC, TIB, TLL, and RPP stand for pulmonary cavity, tree-in-bud sign, total lung lesions, and residual pulmonary parenchyma, respectively.

The radiomic models based on PC, TIB, TLL, RPP, and combined signs exhibited strong performance in both the training and testing sets. The ROC curves for these five models are illustrated in Figure 4. The AUC (95% CI), sensitivity, specificity, accuracy, and balanced F-scores for each model in the training and Testing groups are presented in Table 2.

**Table 2:** Predictive performance of five models on training and testing sets.

Types of radiomics models	Training set					Testing set				
	AUC (95% CI)	Sensitivity	Specificity	Accuracy	F1 score	AUC (95% CI)	Sensitivity	Specificity	Accuracy	F1 score
PC model	0.85 (0.781 ~ 0.92)	0.815	0.614	0.734	0.785	0.85 (0.742 ~ 0.957)	0.821	0.684	0.766	0.807
TIB model	0.808 (0.75 ~ 0.865)	0.753	0.719	0.734	0.711	0.772 (0.675 ~ 0.869)	0.612	0.744	0.674	0.667
TLL model	0.845 (0.793 ~ 0.896)	0.806	0.736	0.766	0.75	0.801 (0.711 ~ 0.891)	0.633	0.721	0.674	0.674
RPP model	0.855 (0.805 ~ 0.905)	0.828	0.719	0.766	0.755	0.817 (0.729 ~ 0.905)	0.755	0.791	0.772	0.779
Combined model	0.886 (0.827 ~ 0.945)	0.785	0.705	0.752	0.791	0.865 (0.764 ~ 0.966)	0.75	0.789	0.766	0.792

PC, TIB, TLL, and RPP stand for pulmonary cavity, tree-in-bud sign, total lung lesions, and residual pulmonary parenchyma, respectively.

In the training set, the AUC values for the PC, TIB, TLL, RPP, and combined model were 0.850 (95% CI: 0.781–0.920), 0.808 (95% CI: 0.750–0.865), 0.845 (95% CI: 0.793–0.896), 0.855 (95% CI: 0.805–0.905), and 0.886 (95% CI: 0.827–0.945), respectively. In the testing set, the AUC values for these models were 0.850 (95% CI: 0.742–0.957), 0.772 (95% CI: 0.675–0.869), 0.801 (95% CI: 0.711–0.891), 0.817 (95% CI: 0.729–0.905), and 0.865 (95% CI: 0.764–0.966), respectively. The combined model demonstrated the best performance in both the training and testing sets.

### 3.4 Model AUC performance

Regarding AUC values, the combined model achieved the highest AUC of 0.886, indicating optimal classification performance on the training data. In the testing set, the combined model again maintained the highest AUC of 0.865, highlighting its superior generalization capability on new, unseen data. Although the PC model displayed a stable AUC of 0.850 in both the training and testing sets, the combined model’s AUC was slightly higher in the testing set, achieving statistical significance (*p <* 0.05). Delong test results revealed significant differences between the combined model and the PC model in the training set (*p* < 0.05), while the testing set difference was not statistically significant (*p >* 0.05); however, the AUC value remained slightly elevated. In comparison to the TIB model, TLL model, and RPP model, the combined model exhibited no significant differences in performance in either the training or testing sets (*p* > 0.05).

### 3.5 Model calibration and decision curve analysis

**Figure 5:**
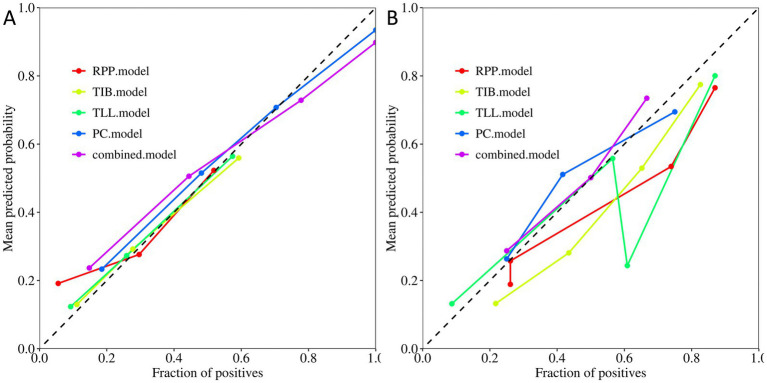
Calibration curves of the PC, TIB, TLL, RPP, and combined model. **(A)** Training Set. **(B)** Testing Set. PC, TIB, TLL, and RPP stand for pulmonary cavity, tree-in-bud sign, total lung lesions, and residual pulmonary parenchyma, respectively.

Figure 5 display the calibration plots for the training and testing sets, demonstrating that the model prediction curves closely align with the ideal calibration line. This suggests that our combined model achieves a high degree of concordance in estimating the probabilities of DS-TB and DR-TB cases, reflecting strong consistency with actual outcomes.

**Figure 6:**
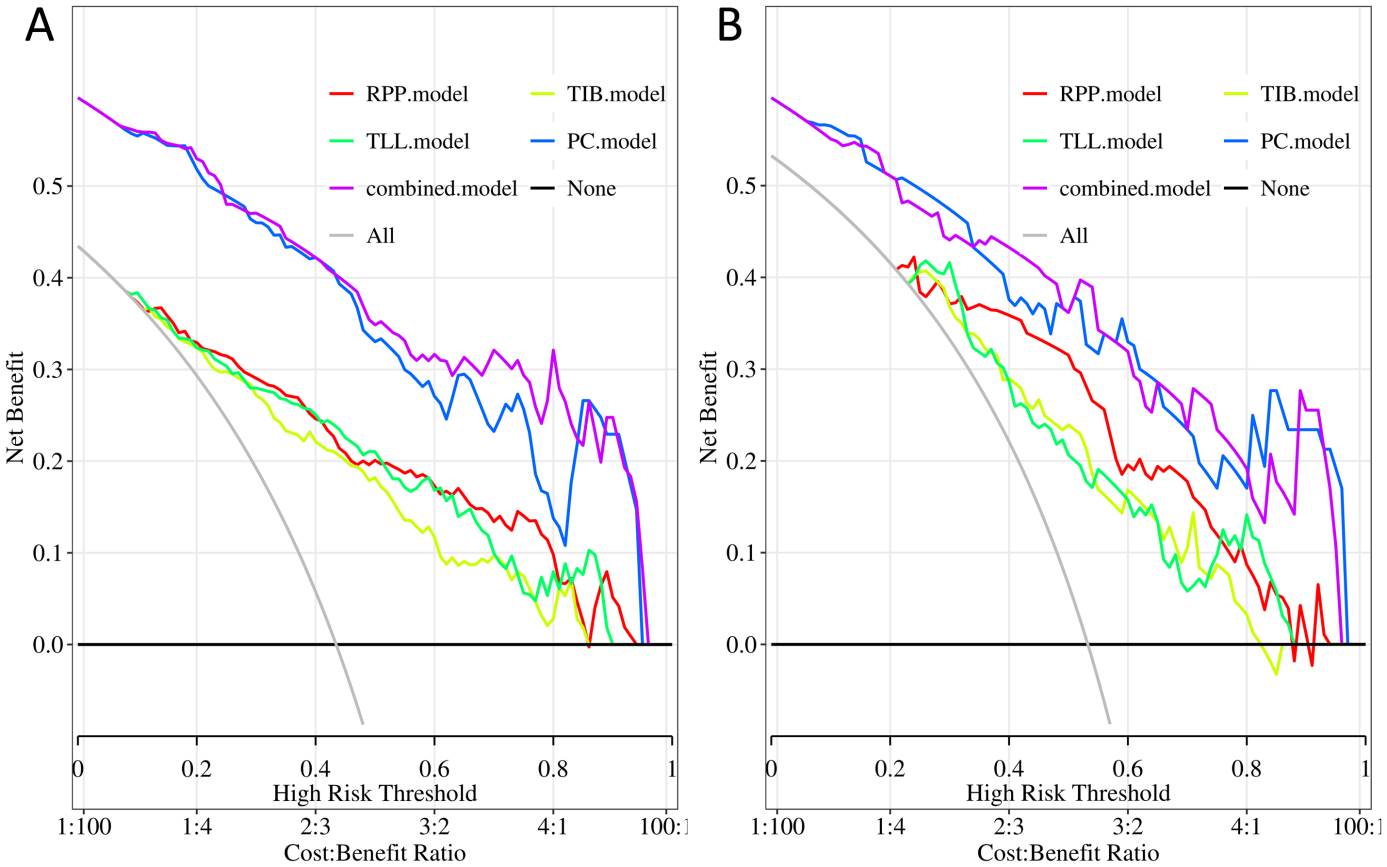
Decision curves of the PC, TIB, TLL, RPP, and combined model. **(A)** Training Set. **(B)** Testing Set. PC, TIB, TLL, and RPP stand for pulmonary cavity, tree-in-bud sign, total lung lesions, and residual pulmonary parenchyma, respectively.

Moreover, decision curve analysis (Figure 6) indicates that, across a broad range of probability thresholds, the combined model delivers optimal clinical net benefits in guiding resistance predictions and optimizing treatment strategies. This emphasizes its significant advantage in medical decision support. Table 2 summarizes the AUC (95% CI), sensitivity, specificity, accuracy, and balanced F-scores for the five models in both the training and testing sets.

## 4 Discussion

In this study, we defined all imaging lesions associated with pulmonary tuberculosis in both lungs as TLL, while normal lung tissue excluding tuberculosis lesions was termed RPP. This represents the first explicit proposal of these concepts and research focus. Previous research in this domain has shown heterogeneity and limitations in quantification, particularly regarding the quantitative analysis of PC, TIB, TLL, and RPP. Against this backdrop, we developed and validated a CT radiomic model incorporating PC, TIB, TLL, RPP, and their combinations to differentiate between DS-TB and DR-TB.

This study collected essential clinical information for all subjects. Statistical analysis revealed significant differences in gender, age, and prior treatment history between the DR-TB and DS-TB groups. The findings indicated a significantly higher proportion of males among DR-TB cases compared to females, and prior treatment history was strongly associated with the development of drug resistance, particularly in adults with a history of retreatment. This is consistent with previous research by Lu et al. (20) on the drug resistance and epidemiological characteristics of multidrug-resistant tuberculosis patients across 17 provinces in China. In this study, the average age of the DR-TB group was older, contrasting with the findings of Khasan et al. (21) on DR-TB patients in Uzbekistan from 2013 to 2018. This discrepancy may be due to differences in sample selection, geographical specificity, and study duration.

The investigation of the association between pulmonary tuberculosis drug resistance and imaging features using radiomics is still in its infancy, limited by the complex and variable appearance of pulmonary tuberculosis on CT images. Multidrug-resistant tuberculosis (MDR-TB) is a special type of DR-TB. Previously, Li et al. (22, 23) reported on the radiomics of MDR-TB patients. In this study, we expanded the study population from special multi-drug resistant patients to general drug-resistant patients, and compared our results with theirs, making our findings more generally applicable.

Cavitary features in tuberculosis lesions are particularly significant, as the bacterial load of *Mycobacterium tuberculosis* (MTB) markedly increases within these PC, while the cavity walls serve as a biological barrier. Together, these elements establish the biological foundation for the emergence of drug resistance in pulmonary tuberculosis (24, 25). Previously, Li et al. (22) developed a radiomics-based predictive model that achieved area under the curve (AUC) values of 0.844 and 0.829 in training and testing sets, respectively, when assessing drug resistance in cavitary pulmonary tuberculosis. In contrast, the model specifically designed in this study for cavitary features achieved AUC values of 0.850 in both the training and testing sets. This finding indicates that the AUC values for the PC model in this study are slightly superior to those reported by Li et al. (22), and the consistency of AUC values across the training and testing sets highlights the model’s robustness and enhanced generalization performance.

In patients with active pulmonary tuberculosis, the TIB sign is a prevalent imaging feature, and the high activity of the disease is often correlated with the high drug resistance characteristics of *Mycobacterium tuberculosis* (MTB) (26). Previously, Li et al. (23) developed a radiomics model for predicting drug resistance in active pulmonary tuberculosis based on the TIB sign, achieving area under the curve (AUC) values of 0.877 and 0.786 in the training and Testing sets, respectively. In contrast, the radiomics model for the TIB sign developed in this study achieved AUC values of 0.808 in the training set and 0.772 in the testing set. While Li et al.’s model exhibited superior performance in the training set, the relatively small decrease in AUC values for this study’s model (from 0.808 to 0.772) suggests a significant advantage regarding generalization and stability. The minimal performance difference observed in the testing set indicates that, in practical applications, this study’s model may offer a more reliable option due to its robustness.

We conducted a detailed comparison of the newly constructed models—namely, the “TLL model” and the “RPP model”—and thoroughly analyzed their key performance metrics against previously reported “PC model” and “TIB model” (22, 23). These metrics included, but were not limited to, AUC values, sensitivity, specificity, accuracy, and balanced F-scores. The findings revealed that both the TLL model and the RPP model demonstrated specific advantages. The TLL model displayed a commendable level of stability and practicality in its overall performance. Despite some decline in various metrics within the testing set, it sustained a strong performance, highlighting its adaptability to new data. In contrast, the RPP model exhibited outstanding AUC values in both training and testing sets, showcasing remarkable classification power, especially with its high specificity and balanced F-scores, indicating its potential in reducing false-positive predictions. In summary, both the TLL model and the RPP model, through optimized feature combinations, not only offer theoretical innovations but also demonstrate enhancements in key performance indicators compared to earlier models in practical applications. This underscores their potential to improve diagnostic efficiency and accuracy.

In conclusion, this study has innovatively integrated four key regions of interest (ROIs): “PC,” “TIB,” “TLL,” and “RPP,” resulting in the construction of a comprehensive combined model. This marks the first proposed model integration strategy in this field. Encouragingly, this combined model demonstrated excellent predictive performance. Analysis of the parameters reveals that the combined model outperformed the other four independent models in overall performance. The strength of the combined model lies in its ability to incorporate multiple features, effectively enhancing diagnostic efficacy. It achieved a high AUC value not only in the training set but also maintained the highest predictive performance in the testing set, demonstrating superior generalizability to new data. The Delong test further confirmed a significant difference between the combined model and the PC model in the training set (*p* < 0.05). Although the difference in the testing set did not reach statistical significance (*p* > 0.05), the numerical advantage was still evident. Calibration and decision curve analyses indicate that the combined model exhibits strong reliability and stability. Based on these findings, the combined model is recognized as the best model due to its exceptional generalization capability and overall diagnostic performance.

To deepen and expand the radiomic analysis of CT imaging features in patients with DR-TB, this study thoroughly investigates both local and overall CT manifestations. In terms of model construction, we initially focused on local imaging markers by developing the “TIB model” and the “PC model.” Following this, we designed the “TLL model” and the “RPP model,” emphasizing global imaging characteristics. Building upon this foundation, we innovatively integrated local and global perspectives to create a comprehensive combined model. This model enhances robustness and accuracy by incorporating diverse imaging features, which is crucial for practical applications. Such a multidimensional feature fusion model can reveal disease characteristics in a more holistic manner, providing precise auxiliary information for clinical decision-making.

Notably, this study has several limitations. First, in applying the model to DR-TB clinical interventions, we have not yet integrated other clinical factors (such as medical history and laboratory test results) to determine a reasonable decision threshold. Maximizing net benefit while considering the feasibility and safety of clinical interventions is our goal, and this is a key issue that needs to be addressed in future research. Second, this study employed a retrospective analysis with a limited sample size, which may have introduced selection bias. These limitations highlight the need for further improvements and validations of the model’s clinical utility in future studies.

## 5 Conclusion

We successfully constructed five radiomic models based on CT imaging data, aimed at effectively identifying patients with drug-resistant tuberculosis. These models underwent rigorous training and validation across five independent cohorts, demonstrating exceptional performance. Importantly, the newly defined and developed RPP model and the combined model showed particularly outstanding performance. This research holds significant value for achieving early non-invasive diagnosis and differentiation of drug-resistant pulmonary tuberculosis, and it has the potential to become a practical, non-invasive diagnostic tool.

## Data Availability

The raw data supporting the conclusions of this article will be made available by the authors, without undue reservation.
